# Protoplast Preparation for Algal Single-Cell Omics Sequencing

**DOI:** 10.3390/microorganisms11020538

**Published:** 2023-02-20

**Authors:** Junran Ye, Cuiqiyun Yang, Luojia Xia, Yinjie Zhu, Li Liu, Huansheng Cao, Yi Tao

**Affiliations:** 1 Division of Natural and Applied Sciences, Duke Kunshan University, 8 Duke Ave, Kunshan 215316, China; 2 Groundwater Provincial Engineering Research Center for Urban Water Recycling and Environmental Safety, Shenzhen International Graduate School, Tsinghua University, Shenzhen 518055, China

**Keywords:** algal protoplast, cell wall, cellulose, enzymatic lysis, bead, single-cell, sequencing, *Chlorella vulgaris*

## Abstract

Single-cell sequencing (SCS) is an evolutionary technique for conducting life science research, providing the highest genome-sale throughput and single-cell resolution and unprecedented capabilities in addressing mechanistic and operational questions. Unfortunately, the current SCS pipeline cannot be directly applied to algal research as algal cells have cell walls, which makes RNA extraction hard for the current SCS platforms. Fortunately, effective methods are available for producing algal protoplasts (cells without cell walls), which can be directly fed into current SCS pipelines. In this review, we first summarize the cell wall structure and chemical composition of algal cell walls, particularly in Chlorophyta, then summarize the advances made in preparing algal protoplasts using physical, chemical, and biological methods, followed by specific cases of algal protoplast production in some commonly used eukaryotic algae. This review provides a timely primer to those interested in applying SCS in eukaryotic algal research.

## 1. Introduction

Since the first case of single-cell RNAseq (scRNAseq) in mouse blastomere and oocyte [1], single-cell sequencing (SCS) has been applied to animal model organisms and recently non-model animals [2,3]. Along the way, new and improved SCS techniques have been developed, e.g., ATAC (Assay for Transposase Accessible Chromatin), single-cell spatial transcriptomics, immune profiling, and integrated multi-omics such as the integrated scRNAseq and spatial transcriptomics and other combinations [4]. SCS integrated with machine learning has become a major revolutionizing force in life science research. With its high throughput and single-cell resolution, SCS plays a vital role in understanding the organization and development of life and its response to environmental changes in time and space in normal or diseased states. More specifically, SCS has demonstrated its power in characterizing cell types and states, gene regulatory mechanisms, normal development, tissue organization, disease pathology, and clinical translational research [5].

The SCS protocol has two prerequisites for study samples: eukaryotic cells without cell walls. As such, its application in plants and algae falls behind since both these groups have cell walls, which, significantly, abolishes the RNA exaction method used for current SCS. For this reason, some approaches are adopted to get around the cell wall obstacle. For example, plant roots, not other tissues, were used in the first plant SCS paper in 2019 because the root cell wall is the thinnest [6]. The need to remove cell walls is because plant cells need to be disrupted to release mature mRNA for SCS, but the rigidity of cell walls significantly reduces cell disruption and leads to low RNA extraction efficiency. Besides plants, algae are another major group with cell walls. The existing mRNA extracting chemistry does not work well on algae, leading to a low number of detected genes (from one hundred to a few hundred), which are only tiny fractions, about 1%, of the genes expressed in bulk transcriptomes (over 38,000 genes and 16,600 genes, respectively) [7,8]. Comparing the performance of RNA extraction from *Chlamydomonas reinhardtii* strains with (CC-4532, CC4533, and CC-1690) and without cell walls (CC-1690), Ma and colleagues found that limited lysis of cells indicated by chlorophyll extraction and lower numbers of sequenced genes were derived from cells with walls. Moreover, overlapping of cell clusters on a UMAP plot was detected in these cells, interfering with the result analysis [8].

**Figure 1:**
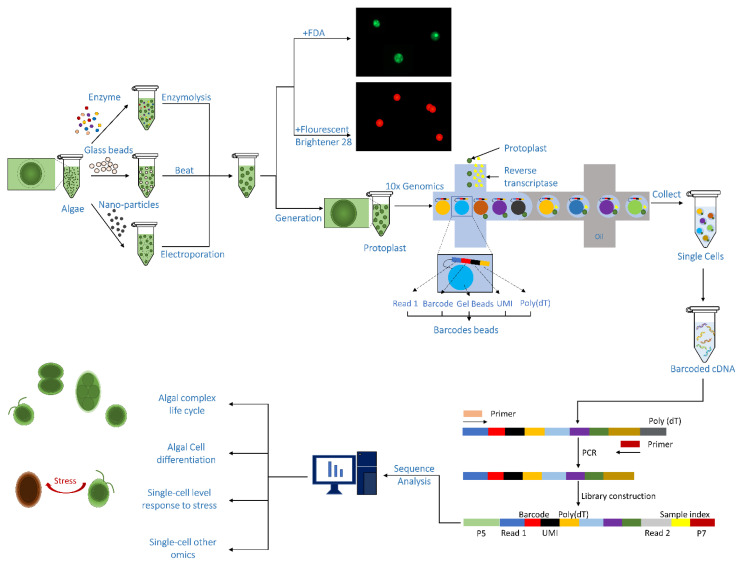
Pipeline of algal protoplasts fed into existing single-cell sequencing pipelines.

This low number of detected genes in current algal SCS presents a major obstacle, which limits its applications in revealing biology [8]. Therefore, this obstacle calls for improved or new methods in either cell preparation or mRNA extraction. One way to improve gene detection in SCS is by making high-quality protoplasts and feeding them to the existing commercial pipelines (Figure 1). Algal protoplasts are cells from which the cell wall is removed, which can then be processed like animal cells for SCS (Figure 1). Fortunately, the methods for generating protoplasts for all major algal phyla have been readily available, as protoplast production and fusion are the starting materials for traditional genetic engineering and state-of-art genome editing with CRISPR (*Clustered Regularly Interspaced Short Palindromic Repeats*) [9,10,11,12].

So far, at least five phyla, eukaryotic Chlorophyta, Rhodophyta, Dinophyta, and Phaeophyta and prokaryotic Cyanobacteria, have had protoplasts successfully produced. Comparatively speaking, there are more species in Chlorophyta than in three other phyla used in protoplasts. The species of the latter phyla only include a few classes. For example, Rhodophyta only has the classes Bangiophyceae [13,14,15], Florideophyceae [13], and Porphyridiophyceae [14]. Most studied brown algae are from the same class Phaeophyceae, e.g., *Dictyopteris pacifica, Scytosiphon lomentaria* [15], *Petalonia fascia* [9], and *Undaria pinnatifida* [16]. In contrast, there are more green algae that have been studied recently, such as *Chlorella protothecoides* [17], *Ulva prolifera* [18], *Haematococcus pluvialis* [19], and *Penium margaritaceum* [20]. In this review, we first examine the cell wall composition of the few best-studied algae, following a summary of the cell wall structure and chemical composition in the best-studied Chlorophyta, then provide a general method for protoplast preparation, and eventually list case studies in these four phyla of algae.

## 2. Cell Wall Structure and Chemical Compositions of Eukaryotic Algae

Among major algal phyla, Chlorophyta is one of the best-studied groups in cell walls. So, we select mainly green algae to demonstrate the cell wall structure and composition. Based on cell wall composition, especially carbohydrates, Chlorophyta is divided into three groups [21,22]: Group 1 (e.g., Prasinophytina and Chlorodendrophyceae) has cell walls made of 2-keto-sugar acid, 3-deoxy-5-O-Methyl-manno-2-octolusonic acid (5OMeKdo), and 3-deoxylxo-2-heptulosaric acid (Dha); Group 2 includes mainly unicellular algae, i.e., the classes Trebouxiophyceae and Chlorophyceae, with cell walls made of monosaccharides such as glucans, algaenans, mannans, and arabionogalactans; Group 3 is composed of marine macroalgae, with building blocks that are mainly sulphated polysaccharides and saccharides such as mannans, xylans, and glucans. In addition, further classification within phyla is based on the presence of glucosamine in the rigid cell wall [23,24], the residue of cell walls after alkali extraction in *Chlorella*. All the above information is summarized in Table 1.

Besides carbohydrates, the algal cell wall also consists of proteins, lipids, and inorganic elements. Their contents vary dramatically between growth states and between species. For example, the cell wall of the well-studied green alga *Neochloris oleoabundans* has ~22% lipids, 31.5% proteins, and 7.8% inorganic matter, which might vary when cells are incubated under different salinity and nitrogen conditions [25]. *Chlorella vulgaris* has 2.46% proteins (dry weight) as well as 15% lipids in its cell walls [26,27], and *Scenedesmus obliquus* possesses 2–16% proteins [26].

The chemical macromolecules are organized into various cell wall structures in algae. Some algae possess only one layer of the cell wall (inner cellulose layer) while others have two layers, namely, a thinner inner carbohydrate layer and an electron-concentrated outer layer in the about 200 nm thick wall. The latter group includes *Neochloris oleoabundans* [22], *Chlorella zofingiensis* [28], and *Chlorella fusca* [29]. Furthermore, the outer layer can be classified into an electron-dense monolayer as in *Neochloris oleoabundans* [22] or a trilaminar layer as in *Chlorella* zofingiensis [28]. Strikingly, a few algal cells even possess three-layered cell walls, including *Penium margaritaceum* [30]: a cellulose inner layer, an interfacing medial layer, and an outer layer consisting of mainly HG-rich lattice complexed with calcium ions. A summary of the cell wall structure is provided in Table 1, which is the basis for generating protoplasts using physical, chemical, and enzymatic methods. Here we provide three typical examples to showcase cell wall composition and structure in green algae.

**Table 1:** Chemical compositions and structures of Chlorophyta cell wall.

Algal Species	Cell Wall Structure	Carbohydrates	Proteins	Lipids	Inorganic Matters	Digestion Enzymes	References
*Neochloris oleoabundans*	an electron-dense outer layer (55 nm) covered with hair-like structures (145 nm) and a less electron-dense internal inner layer (55 nm)	24.3% carbohydrates: rhamnose (~32%), arabinose (<5%), glucosamine (~5%), galactose (~28%), xylose (<5%), mannose (<5%), glucose (~2%), galacturonic acid (<1%), and glucuronic acid (~15%).	31.5% proteins: non-polar amino acid (>67%), polar amino acid (<33%, AA with acidic residue more abundant). Valine, alanine, leucine, glutamic acid, glycine, and aspartic acid are the most abundant	~22% lipids: Tetrahydro-2, 5-dimethyl-2H-pyranmethanol, 5-methyl-3-Hexanol, 1, 3-di-tert-butylbenzene, 2, 4-di-tert-butylphenol, palmitic acid, stearic acid, cis-10-Nonadecenoic acid, 10, 13-Octadecadiynoic acid, methyl ester	7.8% inorganic material: sulphate (~50%), sodium (~30%), phosphate (<5%), potassium (<5%), magnesium (0.19%), and calcium (0.53%) with the former two the most abundant (~80%)	Cellulase, papain, neutral protease	[3,22]
*Chlorella vulgaris*	a phospholipidic acid inner layer	30% carbohydrate: uronic acid (7.7–11.3%) in the rigid wall: glucosamine (100%) in the lysate: rhamnose (~50%), arabinose (~5%), xylose (~10%), mannose (~5%), galactose (~25%), glucose (~5%)	2.46% proteins	15% lipids	-	Acromopeptidase, Cellulysine, Cellulase ONOZUKA R-10, Macerozyme R-10, Chitosanase, Gluczyme, Uskizyme, lytic enzymes	[26,27,31,32,33,34]
*Chlorella* spp.	-	23.4%~28.6% neutral sugars: rhamnose (21.4%~33.9%), fucose (3.8%~6.7%), arabinose (12.3%~19.9%), xylose (5.6%~8.2%), mannose (5.7%~9.5%), galactose (4.0%~5.4%), glucose (15.6%~46.0%), unknown (0.5%~0.7%) 15.4%~19.8% uronic acid 7.0%~16.6% glucosamine	6.4%~10.0% proteins	-	-	-	[35]
*Chlorella vulgaris*	82~144 nm, unilamellar with two layers, i.e., electron-dense outer layer and low-density inner layer	19–27 % monosaccharides: galactose (53–60%), rhamnose (24–26%), xylose (6–8%), glucuronic aicd (5%), glucose (3%) and mannose (2%) glucosamine only in stationary phase (2.3%)	-	-	-	Acromopeptidase, Cellulysine, Cellulase ONOZUKA R-10, Macerozyme R-10, Chitosanase, Gluczyme, Uskizyme, lytic enzymes	[26,27,31,32,33,34]
*Chlorella zofingiensis*	an inner layer and a trilaminar outer layer	in its rigid cell wall: glucose (70%), mannose (30%) in its matrix cell wall: mannose (65%) and glucose (30%), as well as minor amounts of rhamnose and galactose	-	-	-	cellulases, xylanases, amylases enzymes	[28]
*Chlorella homosphaera*	-	in its rigid cell wall; glucose (80%), mannose (15%) in its matrix cell wall: mannose (70%) and glucose (20%), and galactose (10%)	-	-	-	cellulases, xylanases, amylases enzymes	[28]
*Chlorella fusca*	an inner layer and a trilaminar outer layer	Ketocarotenoids and sporopollenin	-	-	-	-	[29]
*Scenedesmus obliquus*	an inner layer and a trilaminar outer layer	neutral sugars (24–74%), uronic acid (1–24%) and glucosamine (0–15%)	2–16% proteins	-	-	-	[26]
*Senedesmus acutus*	-	Fibrallar fraction: mannose (13%) and glucose (87%) Non-fibrillar fraction: rhamnose (23%), arabinose (6%), xylose (21%), galactose (50%)	-	-	-	-	[36]
*Nannochloropsis oculata*	bilayer structure	glucose (68%), 4–8% each of rhamnose, mannose, ribose, xylose, fucose, and galactose	-	-	-	Cellulase, chitinase, chitosanase, lysozyme, lyticase, protease, sulfatase, cellulase Onozuka R10	[37]
*Nannochloropsis gaditana*	bilayer structure	cellulose (75%)	-	-	-	Cellulase, chitinase, chitosanase, lysozyme, lyticase, protease, sulfatase, cellulase Onozuka R10	[37]
*Penium margaritaceum*	An inner layer (consisting of cellulose), an interfacing medial layer, and an outer layer (HG-rich lattice complexed with Ca^2+^)	-	-	-	-	Driselase	[30]
*Botryococcus braunii*	Trilaminar structure with algaenan	a fibrous β-1, 4- and/or β-1, 3-glucan-containing cell wall	-	-	-	-	[37]
*Desmodesmus* spp.	an outer cell wall layer with net-like structure	-	-	-	-	-	[38]
*Crypthecodinium cohnii*	outermost membrane, outer plate membrane, cytoplasmic membrane, and thecal plates	13% monosaccharides: glucose (86%), galactose (14%), and mannose (0.2%)	-	-	-	Thermostable α-amylase, amyloglucosidase, subtilisin A protease	[33]

Modified from [39].

### 2.1. Cell Wall Structure of Haematococcus pluvialis

**Figure 2:**
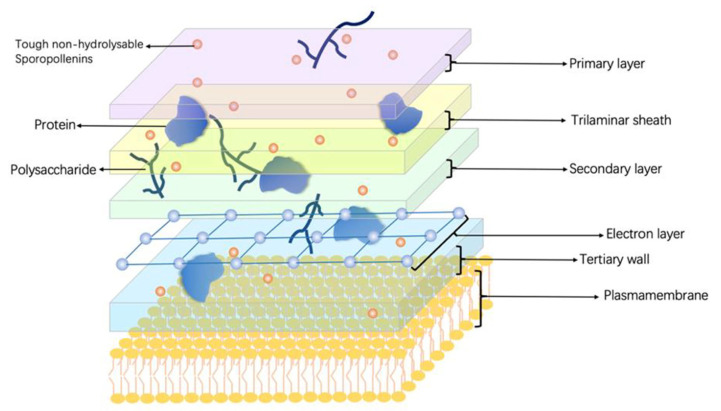
Cell wall structure and chemical compositions of *Haematococcus pluvialis*. *H. pluvialis* cells are morphologically classified into four types: flagellated cells, palmelloid cells, intermediate cells, and cysts. During the cyst period, *H. pluvialis*’s cell wall has a multilayer structure, including a primary layer on the outside, followed by a trilaminar sheath, a secondary layer, a tertiary wall, and an electron later located between the secondary and tertiary wall. Polysaccharides, proteins, and tough non-hydrolysable sporopollenins are the main constituents of the cell wall.

*H. pluvialis* is a unicellular freshwater green alga that produces many bioactive compounds, including astaxanthin and carotenoids. Its morphology changes with growth conditions and the stages of the cell cycle, which are morphologically classified into four types: flagellated cells, palmelloid cells, intermediate cells, and cysts [40,41] (Figure 2). Among them, cysts are the most studied. The cell wall of *H. pluvialis* during the cyst period is composed of five layers: a primary layer on the outside, followed by a trilaminar sheath, a secondary layer, a tertiary wall [21], and an electron later located between the secondary and tertiary wall [42]. Among all the layers, the electron layer is electron-dense, thin, and translucent. It is determined that polysaccharides, proteins, and tough non-hydrolysable sporopollenins [43] are the main constituents of the cell wall. The proteins in the cell wall proteins are mainly cell wall-modifying enzymes [40].

### 2.2. Cell Wall Structure and Chemical Composition of Chlorella vulgaris

**Figure 3:**
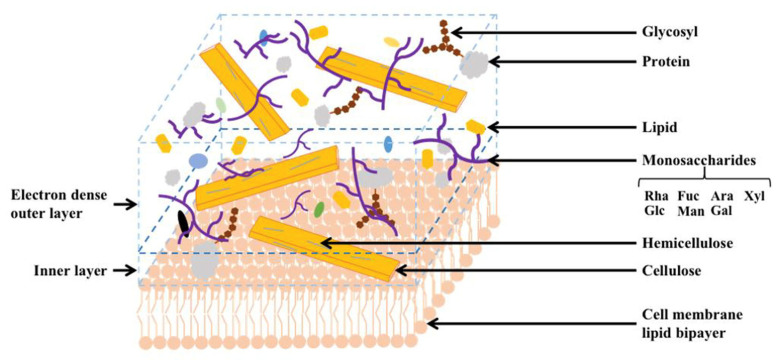
Cell wall structure and chemical compositions of *Chlorella vulgaris*. *C. vulgaris* cell wall has a unilamellar structure, consisting of an inner layer with an electron-dense outer wall and a low-density layer, and an outer layer with hairlike fibers. The cell wall of *C. vulgaris* is mainly composed of cellulose, hemicellulose, monosaccharides, lipid, protein, and glycosyl. As a glucosamine-type species, glucosamine is the main constituent of the cell wall of *C. vulgaris*, and proteins constitute about 20% of the cell wall.

Transmission electron microscopy reveals two different layers in the unilamellar cell wall of *C. vulgaris*: the inner layer consisting of an electron-dense outer layer (also referred to as hairlike fibers) and a low-density layer [33] (Figure 3). The electron-dense thin layer is about 17~20 nm thick [44]. Hairlike fibers are located at the surface of the cell wall. Relatively long straight microfibrils from the extracted cell wall are layered, likely made of mannoglucan, with interfibrillar material not observed as in the other study [45]. Earlier studies postulated multiple network-like structures may exist within the *C. vulgaris* cell wall. The chitin-like glycans discovered might contribute to the resistance to acetolysis, and a microfibrillar layer composed of glucosamine after maturation is recently observed [45,46].

Glucosamine composition is the distinguishing compound between the glucan-mannan type and glucosamine-type in chlorococcal algal species [31,47]. Glucosamine is the main constituent in *C. vulgaris* cell walls, indicating that it is a glucosamine-type species. However, glucosamine existence is detected only in the stationary phase, accounting for 2.3% of dry mass [33]. The matrix sugar composition and the major sugar composition are dominated by rhamnose and galactose [33,47]. In the extracellular polysaccharides (EPSs) of *C. vulgaris,* glucose (about 70–75%) is the most abundant sugar during the exponential phase, followed by galactose and a small amount of mannose, whereas during the stationary phase, galactose is the most abundant (about 65–75%), followed by glucose, arabinose, mannose, and glucuronic acid [33].

Besides simple sugar composition within cell wall polysaccharides, non-fiber carbohydrates such as beta-1,3-glucan are also identified in the *C. vulgaris* cell wall structure [48]. Additionally, *C. vulgaris* contains various biopolymers in its cell wall including a higher proportion of polyamides, which facilitate the cross-linking of the cell wall polymers and sporopollenin, conferring cell wall rigidity and enzymatic digestion resistance [31,32]. The cell wall is composed of about 20% of the total protein component with 50% of it located in the cell matrix [31]. However, the exact protein components are unknown.

### 2.3. Cell Wall Structure and Chemical Composition of Chlamydomonas reinhardtii

As a model organism, the cell wall structure of *C. reinhardtii* is well understood. Its cell wall has five layers, which are different in structure and chemical composition, namely, W1, W2, W4, W6, and W7, from innermost to outermost. The five layers differ from each other in structure and chemical composition. W3 and W5 are not considered physical layers but interlayer spaces between W2 and W4 and W4 and W6, respectively [49]. W2, W4, and W6 constitute a prominent central triplet. A loose web of fibers populates the innermost W1, connecting the plasmalemma and the central triplet [50]. W1 appears to be an open trabecula because of its anastomosing fibers of various caliber and varying depths. W2 consists of a dense network of anastomosing fibers organized into a tighter “weave” than W1. Within W2, thinner fibers interconnect the thick fibers, which then lie parallel to the cell surface. Both W1 and W2 are capable of resisting extraction by chaotropic agents. Additionally, W2 contains major substrate hydroxyproline-rich glycoproteins, which can be degraded by the wall-degrading enzyme glysin. W4 lacks fibrous elements but instead contains 14 nm granules densely aligned in parallel to the membranes in loosely associated rows. The W4 granules have a specific affinity for the W6 components, constituting the central layer of bilaminar ‘crystals’ (W6-W4-W6) when assembled in vitro. W6 is an asymmetric bilaminar matrix, which has inner (W6A) and outer (W6B) sublayers. Inside, the W6A is a dense grid-like mesh network of parallel fibers connected with thin cross-fibrils, and W6B is an open weave resembling a polygonal lattice. W7 is a loosely organized reticulum, containing a variable number of anastomosing fibers resembling those of W1. In addition, W7 can be extracted by chaotropic agents, but its constituent molecules show no tendency to assemble in vitro.

This multilayered cell wall is carbohydrate-deficient and largely composed of 25–30% glycoproteins that contain a large proportion of hydroxyproline [50]. Galacto- and arabinofuranose are also detected in the cell wall glycoprotein structure [51]. These proteins form two major domains that are separate from each other. One outer domain contains about twenty kinds of different proteins, which are held together by noncovalent interactions [52]. This domain constitutes the mass of the cell wall and can be extracted with chaotropic agents, such as boiling in SDS-dithiothreitol [50], showing that disulfide linkages are critical to the wall integrity [52]. The other domain makes a wall-shaped complex consisting of a few kinds of proteins [52]. This domain provides the most mechanical support for cell integrity and shape maintenance and is rich in sarkosylurea-insensitive and SDS-dithiothreitol-sensitive linkages, and is therefore insoluble in chaotropic agents [50].

## 3. Methods for Preparing Algal Protoplasts

Protoplast preparation and fusion is a standard genetic engineering procedure developed in the early 1960s for plants and algae, which is now integrated with state-of-art genome editing CRISPR [11,12,53,54,55]. In the early stage, this method is based on enzymatic lysis of cell walls and quickly applied to all major algal phyla, including Blue-green algae (cyanobacteria) and green, red, and brown algae [10]. Recent developments also use physical approaches, mainly cell disruption with glass beads [56] and nanoparticles [57]. Here we summarize physical, chemical (chelating), and enzymatic approaches in representative species.

### 3.1. Physical Methods

#### 3.1.1. Protoplast Preparation with Nanoparticles

This method has proved especially efficient for yeasts *Saccharomyces cerevisiae* and *Pichia pastoris*, which show intrinsic lytic activity [57]. This method could be applied to algal species with appropriate modification. Briefly, iron oxide (Fe_3_O_4_) magnetic nanoparticles (MNPs) were prepared with FeCl_3_ and FeCl_2_ solution, whose Fe^2+^/Fe^3+^ ratio under pH 10 is 1:2. Homogeneous cell suspension is obtained after a cold wash of centrifugation pellet and resuspension with PBS (pH 7.5) and gentle stirring. An amount of 1.8 mg Fe_3_O_4_ MNP was added to 200 μL yeast cell suspension, and the mixture was shaken for an arbitrary duration between 0 and 2 h at 200 rpm on a rotary shaker at 25 °C. Fe3O4 was removed by magnetic separation. Multiple examinations showed that only the cell walls were disrupted while the cell membranes were intact, generating protoplasts. The efficiency was close to physical disruption (e.g., vigorous vortexing, grinding, blending), mechanical disruption (e.g., high-pressure homogenization, sonication), and enzymatic lysis. The resulting protoplasts show normal regeneration in downstream manipulations. A recent study also demonstrates Fe_3_O_4_ MNP’s potential in identifying protoplasts generated after enzymatic or physical treatment, which is named magnetic immobilization [56].

#### 3.1.2. Protoplast Preparation with Glass Beads

Glass beads of 1.0 mm in diameter (Sigma, St Louis, MO, USA), were washed and baked at 180 °C for 2–3 h. *Haemetacoccus pluvialis* cells of the logarithmic phase were harvested by centrifugation, washed with 25 mM phosphate buffer (pH 7.0) containing 0.6 M D-mannitol, and re-suspended with this medium at a concentration of 2 × 10^8^ cells/mL. An amount of 200 mg of dry glass beads was added to 2 × 10^8^ cells in a 1.5-mL Eppendorf tube and shaken at 1500 rpm on a vortex agitator for 30 s. Vortexing for 30 s and 200 mg of dry glass beads produced the best result [55].

Physical methods, though not common, are gaining popularity and attracting researchers’ interest. Existing physical methods use nanoparticles and glass beads for cell wall disruption. There are both strengths and limitations to this method. Physical methods are of high efficiency, as the time needed to isolate protoplast is shorter than traditional enzymatic methods. It has been verified that around 80% of the yeast cells were lysed within 10 min by the Fe_3_O_4_ magnetic nanoparticles, while around 70% was lysed by zymolyase after 2 h under the same conditions [57]. Additionally, as nanoparticles and beads can work in a wide range of temperatures, they can be used at low temperatures to reduce mRNA degradation. Moreover, as this approach is not selective, it is possible for it to be developed into a universal protocol for protoplast isolation of different algal species and simplify the whole process. However, the physical methods have some potential problems. In particular, the size of the beads usually does not work well with algae cells. Ideally, the beads should be equal to or smaller than the cells, but these sizes are not easy to manufacture. This size discrepancy leads to a long shaking time and generates a lot of heat which unfavorably increases the temperatures of the algal culture. Additionally, as algal cells are heterogeneous in the fluid culture medium, it is possible that some cells have become protoplasts while others are still intact with cell walls, which makes the separation of intact cells from protoplasts hard [55].

### 3.2. Enzymatic Lysis

**Figure 4:**
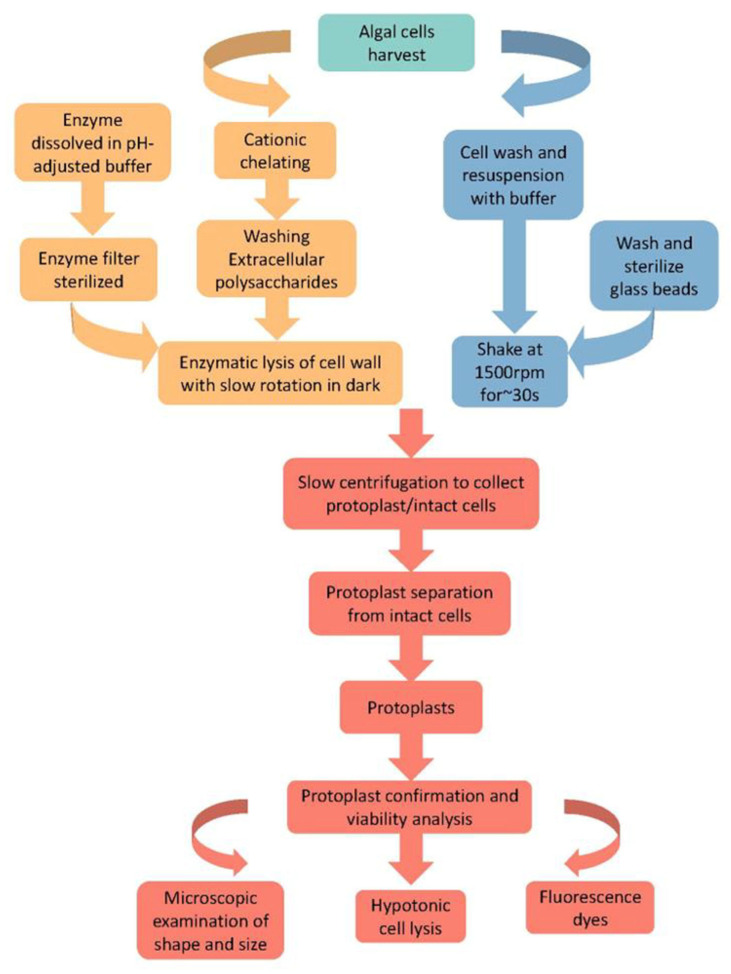
General protocols for protoplast isolation from algal cells. The protocol is mainly composed of five steps, including enzyme solution preparation, cell preparation, chelator treatment, enzymolysis, protoplast collection, and protoplast confirmation and viability analysis.

Cell wall lysis with appropriate enzymes is a long-established approach for preparing algal protoplasts. The general steps in enzymatic lysis are summarized in Figure 4. As cell wall structures and their composition differ between algae, appropriate enzymes should be used to achieve optimal lysis. It is noteworthy that to enhance enzymatic lysis, nonspecific chemical disruption of the bonds stabilizing cell wall components is also used to treat the cells. For instance, the use of cation chelator EDTA (Ethylenediamine Tetraacetic Acid) and calcium chelator EGTA (Ethylene Glycol Tetraacetic Acid) [58,59]. Here we introduce the specific steps (Figure 4).

#### 3.2.1. Sample Collection

Most enzymatic studies show that it is easiest to make protoplasts from the cells of the logarithmic growth phase [34,60,61,62]. This may be due to the fact the cells are growing and the cell wall is weak during depolymerization and susceptible to enzymatic attack [34].

#### 3.2.2. Pre-Processing

Cells are preprocessed mainly to wash and reduce excess extracellular polysaccharides (EPSs) or gel [61], besides reducing contaminations for natural samples. This is achieved mainly by pelleting cells down and vortexing them in fresh media.

#### 3.2.3. Enzyme Solution Preparation 

For cell wall digestion, cellulase (cellulase onozaka R-10, cellulysin), driselase, pectinase (pectate lyase, pectolyase Y23) and macerozyme R-10 are the commonly used enzymes for freshwater algae. Moreover, different algal species need additional enzymes. For instance, green alga *Chlorella* can be treated with snailase [17] because it is part of the snail forage; marine algae need alginate lyase [15,60] because their cell wall contains alginate or alginic acid. Similarly, macerase [63], pronase [62], and proteinase K [62] are also needed for some algae. These enzymes are usually prepared in buffers, such as MES buffer [64] and phosphate buffer [61] adjusted to the appropriate pH. Before application, enzyme solutions are sterilized by passing through 0.22 μm membrane filters.

#### 3.2.4. Protoplast Isolation with Chelator Pre-Treatment 

The binding of cations (e.g., calcium) with macromolecules stabilizes cell walls (e.g., [30,37]). Therefore, the disruption of these bonds with chelators such as EDTA or EGTA has proved useful in producing protoplasts [54,61]. EDTA (ethylenediaminetetraacetic acid) or EGTA (ethylene glycol-bis(β-aminoethyl ether)-N,N,N′,N′-tetraacetic acid) adjusted to appropriate pH (e.g., 6) is mixed with cells, which are rotated gently [15,61,65]. Besides the chelator solution, osmotic stabilizers enhance the efficiency of protoplast generation significantly [61]. For example, 2~5% D-mannitol as the osmotic stabilizer helps maintain the dehydration status of algal cells and facilitates cell wall digestion [61,63].

#### 3.2.5. Separation of Protoplast and Undigested Cells 

One common separation method is membrane filtration, taking advantage of shape/size differences between protoplasts and undigested cells. For example, polyester (PET) mesh filters of 40 μm, 15 μm, and 10 μm are used in this procedure [66]. The undigested cells still having cell walls are left on the mesh surface while the protoplasts pass through the mesh successfully. Protoplasts are resuspended with mannitol with gentle rotation and collected by centrifugation.

#### 3.2.6. Confirmation of Protoplasts 

Shape and size are the most apparent characteristics differentiating protoplasts (smaller and spherical) from undigested cells (larger and various shapes) [65]. Additionally, the hypotonic test is another method to check for protoplasts, which burst in hypotonic solutions while the undigested cells are intact. Another straightforward way is by visualizing protoplasts with staining. Fluorescence dyes include Calcofluor White M2R [15,34,67] as a fluorescent brightening agent for cellulose in cell walls. Other types of dyes can reach cytosol or cell membranes that are only accessible in protoplasts. For example, fluorescein diacetate [15,54] can penetrate cell membranes and produce fluorescein in cells with an active metabolism; Di-4-ANEPPDHQ [61] shows fluorescein when the charges changed in the surrounding environment; FL C11-Phosphocholine [61] labels phospholipids; and FL C5-Ceramide [61] labels sphingosine.

There has been some important progress in algal cell wall digestion with various types of enzymes [15,17,60,62,63]. Among them, cellulase and pectinase are the most widely used and proved to be the most effective, while macerozyme and driselase as well as alginate lyase could be added as needed. Although enzymatic methods have been extensively studied and successfully used for protoplast preparation for years, the method has limitations and could be optimized. First, the type and amount of enzymes should be experimentally predetermined for different algae, which have different cell walls and chemical compositions. This is time-consuming. Additionally, the enzymes selected for cell wall degradation may further digest components on or in protoplasts. For example, as some algal cell walls are rich in protein, protease is added in protoplast preparation, which will degrade the cell membranes and destroy protoplasts. Moreover, the enzymolysis of the cell wall can also be quite time-consuming due to low catalytic efficiency, particularly at low temperatures. It usually takes hours to degrade the cell wall, and the exact experiment time varies among different algae. For example, it takes over 3 h for *Chlorella vullgaris* protoplast preparation [34] and even 15 h to degrade the *Enteromorpha intestinalis* cell wall [54]. Moreover, during the whole enzymolysis process, the temperature of the surrounding environment needs to be strictly controlled as different enzymes have different optimum working temperatures.

#### 3.2.7. Representative Cases of Algal Protoplast Preparation 

A variety of algal protoplast isolation strategies are described species by species of several phyla briefly as follows.

*Chlorella vulgaris* (Chlorellaceae, Chlorophyta) is a microalga. The enzymatic method for preparing *C. vulgaris* protoplasts has been described, which we summarize here. Algae were grown in Myers-4N medium at 25 °C with a photon flux density of 250 µmol/m^2^s and 1.3% CO_2_ in air. At the L_2_ stage, the intermediate stage during the ripening phase of the cell cycle, algae cells were collected [68]. The enzyme solution was prepared with homogenates of *C. vulgaris* and rotifer, and both homogenates contained lytic enzymes. To prepare algal homogenates, *C. vulgaris* cells at the L_4_ stage, which is the stage just before cell division, were collected at 4 °C and washed with distilled water. The suspension was then homogenized with glass beads of 0.5 mm in a reciprocal shaker and filtrated. All the processes were performed at 4 °C. The rotifer homogenate was prepared by homogenizing frozen rotifer in phosphate buffer with 1 mM PMSF and mixed with ammonium sulfate until reaching 80% saturation. The mixture was then pelleted and then dissolved in 50 mM sodium phosphate buffer with 1 mM PMSF. Besides the two homogenates, other commercially available enzymes were also added, including acromopeptidase, cellulysine, cellulase, macerozyme, chitosanase, gluczyme, pectinase, and uskizyme, and the enzymes were dissolved in sodium phosphate buffer. After the enzyme solution was prepared, algal cells were resuspended in sodium phosphate buffer and then mixed with enzyme solutions and incubated at 30 °C for 3 h in the dark to induce the appearance of protoplasts. Eventually, the protoplast generation was examined by adding distilled water and counting unburst cells or fluorescence microscopy by staining the cell wall with Fluorescent Brighter 28 (M2R).

*Micrasterias denticulata* (Desmidiaceae, Charophyta) is a microalga. Protoplasts were prepared by a combination of chemical and enzymatic methods. Cells from 100 mL of culture were collected by centrifugation at 5000 rpm, washed with distilled water, and vortexed for preparation. Then, cells were incubated with 3–9% mannitol, 2–6% Cellulysin, and 4 mM calcium chloride at 22 °C in darkness overnight. All the ingredients are prepared with desmidian medium. Then, the mixture was maintained at 37 °C for 2 h. The protoplast can be observed under a light microscope [65].

*Haematococcus pluvialis* (Haematococcaceae, Chlorophyta) is a microalga [62]. *H. pluvialis* protoplasts were prepared with an enzymatic method. Cells were cultured in B/5 medium in a 12 h light: 12 h dark light cycle with 50 rpm rotation at 20 °C for 6–7 days. The enzyme solution was prepared by mixing 0.06% Proteinase K and pronase, together with 0.2 M sorbitol and mannitol (1:1) in B/5 medium and filtering through 0.45 um cellulose nitrate membranes. Next, 0.1 mL of cells was mixed with 0.2 mL of triethanolamine buffer (50 mM, pH 7.8), 0.4 mL of the enzyme solution, and 0.3 mL of deionized water, which was subjected to reciprocal shaking at 100 strokes/min for 1 h at 35 °C. The resulting protoplasts were examined with the hypotonic treatment of digested and undigested cells.

*Draparnaldia* sp. (Chaetophoraceae, Chlorophyta) is a macroalga. *Draparnaldia* sp. protoplasts were also prepared with an enzymatic method. An amount of 3–4 g of fresh biomass was cut using forceps and placed in a Petri dish (100 mm × 20 mm), before being resuspended in 12 mL 0.5 M mannitol and sealed with Parafilm for incubation on a rotator (70 rpm shaking) for 35–40 min at room temperature. An enzyme solution of 2.5% of Driselase was prepared by dissolving 0.25 g Driselase in 10 mL of 0.5 M mannitol solution, vortexing, and wrapping it with aluminum foil. Next, the enzyme solution was cooled to 4 °C, centrifuged at 2500× *g* for 10 min, and sterilized through a 0.2 μm filter. The algal pieces were then mixed with enzyme solution and incubated with shaking at 30–40 rpm and room temperature for 45–60 min. Then, protoplasts and unbroken cells were separated through polyester (PET) mesh filtration (40 μm pore size) followed by rising with mannitol. The retained materials were further filtered through the PET mesh with small pore sizes (15 and 10 μm). Finally, protoplasts were centrifuged and resuspended with 5 mL 0.5 M mannitol by gentle rotation. The process was repeated three times to harvest protoplasts. The protoplast generation was confirmed by OD_750_ measurement and M2R staining [66].

*Mougeotia* sp. (Zygnemataceae, Charophyta), *Ulothrix fimbriata* (Ulotrichaceae, Charophyta), and *Klebsormidium (Hormidium) flaccidum* (Klebsormidiaceae, Charophyta) are filamentous macroalgae [63]. Protoplasts were generated enzymatically. Algae were grown in Pocock’s medium at 20 °C with a 16 h:8 h light-dark cycle. Cells collected at the beginning of the dark cycle were plasmolyzed in a solution of 0.3 M mannitol, 0.3 M sorbitol, 2 mM CaCl_2_·2H_2_O, and 2 mM NaH_2_PO4. Then, the enzyme solution was prepared by dissolving 2% (*w*/*v*) Cellulysin and 0.1% (*w*/*v*) Macerase in 25 mL of plasmolysis solution. Then, cells and enzyme solution were mixed and incubated for 1–4 h. The mixture was further possessed with 50 μm nylon mesh filtration and centrifugation at 500× *g* before washing the pellet three times with plasmolysis solution. Protoplasts were harvested by centrifugation at 100–300× *g*. Protoplast generation was examined by M2R staining under the fluorescence microscope.

*Scenedesmus obliquus* (Scenedesmaceae, Chlorophyta) is a microalga. The enzymatic method for protoplast preparation is slightly different from those used for other algae. *S. obliquus* cells were collected by centrifugation at 2000× *g* and resuspension for 30 s in BG11 medium. After that, the collected *S. obliquus* were incubated for ten to fourteen days at 25–27 °C, in a light cycle of 14 h:10 h with a light intensity of 50 μmol·m^−2^·s^−1^ until they reached the log-phase growth period. The enzyme solution was prepared with commercial enzymes and *Daphnia magna* culture fed with algal cells. The *D. magna* culture was mixed with distilled water with a pH adjusted to 7.8 with NaOH and HCl. The *D. magna* solution was incubated at 25–27 °C in a light cycle of 14 h:10 h with 50 μmol·m^−2^·s^−1^ light density. The prepared *D. magna* solution was then mixed with cellulase, pectase, mannitol, and CaCl_2_. The enzyme solution was then mixed with resuspended algal cells and the enzymolysis was maintained at 29–31 °C with shaking at 50–80 rpm for 8–12 h in darkness. The protoplast generation was examined in the reduction in cell numbers after hypotonic treatment [69].

*Dictyopteris Pacifica* (Dictyotaceae, Ochrophyta), *Scytosiphon lomentaria* (Scytosiphonaceae, Ochrophyta), *Sphacelaria Phaeophyceae* (Sphacelariaceae, Gyrista) are macroalgae [60,70,71]. Their protoplasts were all prepared enzymatically. For pre-possessing, the monosporangial germlines separated from the original sporophytes were cultured with Provasoli-enriched seawater (PES) medium until filaments were produced. *S. lomentaria*’s thalli were grown in glass bottles filled with natural seawater and Procasoli-enriched medium (PES) [60]. The buffer used for the enzyme solution contained sea salt elements. For the enzyme solution, alginate lyase and other enzymes were dissolved in the buffer and then mixed with the algal culture. After digestion, protoplasts were purified by passing through a metal sieve (100 μm pore size) to remove non-digested debris. Undigested cells were separated from protoplasts through multiple filtrations, centrifugation, and resuspension. *D. Pacifica* protoplast generation was confirmed by red chlorophyll autofluorescence and M2R staining, while *S. lomentaria*’s protoplast generation was confirmed by microscopic observation [15,70].

Macrophytic marine algae, e.g., *Ulva conglobate* (Ulvaveae, Chlorophyta), *Ulva Fasciata* (Ulvaceae, Chlorophyta), *Ulva Lactuca*, *Ulva Pertusa* (Ulvaceae, Chlorophyta), and *Monostroma oxyspermum* (Monostromataceae, Chlorophyta), have their protoplasts prepared as follows. In prepossessing, young vegetative portions of thallus were thoroughly cleaned with a brush in filtered autoclaved seawater (ASW) under a microscope and then chopped into small pieces (1 mm). The enzyme solution was prepared by mixing Cellulase Onozuka R-10 (2%) with pre-cooled (4 °C) de-ionized water, NaCl (1%), and 0.8 M mannitol, followed by centrifugation at 10,000× *g* for 20 min at 4 °C. The algal pieces were transferred to a 60 mm × 15 mm Petri dish containing 5 mL of enzyme solution and incubated on a rotary shaker (40–50 rpm) in the dark at 20 ± 1 °C for 2 h. Next, the contents of the Petri dish were passed through a nylon mesh (25–30 mm pore size). The suspension was centrifuged at 120× *g* for 5 min, and half of the supernatant was replaced with the same volume of ASW for diluting the osmoticum and enzyme in the suspension. This step was repeated twice. The protoplast generation was confirmed by microscopic observation [64].

*Monostroma latissimum* (Monostromataceae, Chlorophyta) is a macroalga. The enzymatic method was applied for preparing its protoplasts. For the enzyme solution, Cellulase Onozuka R-10 (4%) and Macerozyme R-10 (2%) with 10 mL of 1.2 M sorbitol were mixed and the pH was adjusted with Na_2_HPO_4_-NaH_2_PO_4_ buffer. Then, the solution was centrifuged at 10,000× *g* and 8 °C for 10 min and further sterilized with a 0.2 mm disposable syringe filter. Secondly, the cells were cut into 0.5–1 mm^3^ and incubated with 10 mL enzyme solution on sterile 50 mm × 80 mm disposable plastic flasks (40 mL, Falcon). The culture was placed on an orbital shaker (50 rpm) under 24 °C in dark conditions for 6 h. After digestion, protoplasts and unbroken cells were separated by filtering the contents through a 59-μm nylon mesh, then layered over a 35% (*w*/*v*) density buffer solution. Eventually, the products were harvested by centrifugation at 100× *g* for 30 min. The generation of protoplasts was confirmed by Calcofluor White staining, while protoplasts without cell walls were red under microscopic observation [72].

*Chlorella Protothecoides* (Chlorellaceae, Chlorophyta) is a microalga [17]. *C*. *Protothecoides* protoplasts were prepared by the enzymatic method. Firstly, *C. protothecoides* cells were collected at their log phase by centrifugation at 900× *g* for 5 min. The pellet was suspended in 25 mM Tris buffer (pH 6.0) and 0.6M D-mannitol. The enzyme solution was prepared by dissolving Cellulase R-10 and Snailase in Tris buffer and D-mannitol. The suspension was further mixed with enzyme solution until the final concentration of Cellulase R-10 and Snailase reached 2% and 1%, respectively. Next, the mixture was incubated at 30 °C for 10 h. The product was harvested by centrifugation at 300× *g* for 2 min, resuspending the deposit with a 1.5 M sugar solution. The protoplast formation was checked by counting the disrupted cells of 0.1 mL supernatant added to 0.9 mL deionized water.

*Ulva pertusa* (Ulvaceae, Chlorophyta) is a macroalga. *U*. *pertusa* protoplasts were prepared by the enzymatic method. Firstly, the unialgal culture of *U. pertusa* was prepared by subculturing the algae every month with a PES medium [67]. The female gametophytes of this specie were used as samples. Secondly, the enzyme solution was prepared by dissolving Cellulase Onozuka R-10 (2%), Macerozyme R-200 (0.1%), and Abalone Acetone Powder (AAP) (5%) in 1.2 M sorbitol-MES buffer under PH 5.5. Next, cells were maintained in a sorbitol-MES buffer solution for 1 h. Thirdly, 250 mg cells mixed with 2.5 mL enzyme solution were incubated on a reciprocal shaker (30 strokes/min) at 20 °C for 5 h. Fourthly, undigested cellular debris was separated by 40 mm nylon mesh filtration. Protoplasts were further collected by centrifugation at 50× *g* for 10 min. Finally, the mixture was washed with sorbitol-MES buffer solution several times and diluted on PES medium at 2 mL/h for 5 h. To check the remaining cell walls, Calcofluor White M2R staining and a fluorescent microscope were used.

*Gracilaria dura* (Gracilariaceae, Rhodophyta) is a macroalga. The enzymatic method for protoplast preparation is described below. Algae were collected and cultured under cool white fluorescent lights at 15 μmol photons m^−2^ s^−1^ with a 12:12 h light/dark photoperiod. The enzymatic solution was prepared, consisting of several commercial enzymes for protoplast isolation, including cellulase Onozuka R-10, macerozyme R-10, agarase, papain, and pectolyase. All the enzymes were dissolved in 60% seawater, consisting of 30‰ seawater diluted with Milli-Q water and 25 mM MES adjusted to pH 6.0. The enzymatic solution was first centrifuged at 10,000× *g* for 20 min at 4 °C to remove the insoluble materials and then mixed with algae for protoplast preparation. The mixture was incubated on a rotary shaker (50 rpm) for 6–8 h in the dark at 25 °C. The protoplast yields were estimated by counting the cells using a hemocytometer under an inverted microscope [13].

*Hecatonema terminale* (Chordariaceae, Ochrophyta) is a macroalga. Protoplasts were prepared enzymatically as summarized below. Filaments of *H. terminale* were cultured under a 14:10-h light/dark photoperiod with a light intensity of 40 μmol photons m^−2^ s^−1^ at 20 °C. Then, the enzymatic solution was prepared by dissolving cellulase Onozuka RS and R-10, macerozyme R-10, and alginate lyase. Algae were then mixed with the solution and shaken at 70 rpm, 15 °C for 15 h in the dark for incubation. After that, the protoplasts were filtered by a 25-μm nylon mesh to remove undigested filaments and concentrated by centrifugation at 100× *g* for 10 min. Protoplast generation was confirmed by M2R staining and examination with an inverted microscope equipped with a 360/40-nm emission filter and a 425-nm suppression filter [73].

*Petalonia fascia* (Scytosiphonaceae, Ochrophyta) is a macroalga. Protoplasts were generated by the enzymatic method. Prior to enzymatic digestion, algae were first treated with a calcium-chelating solution with EGTA-Na_4_ as the calcium chelator for 20 min. Then, cellulase Onozuka RS, alginate lyase, and driselase were mixed as the enzymatic solution. After filter sterilization, the enzymatic solution was then mixed with alga and incubated at 20 °C with shaking at 70 rpm in the dark. Protoplast generation was assessed by M2R staining under the fluorescence microscope [9].

*Porphyra nereocystis* Anderson (Rhodophyta, Bangiales) is a macroalga. Protoplasts of *P. nereocystis* Anderson are prepared by two-step digestion by commercial enzymes, first with papain (PAP) and later abalone acetone (AAP). To prepare the enzyme solution, PAP (10% *w*/*v*) and AAP (2% *w*/*v*) are dissolved in sterile seawater, respectively. Porassium dextran sulfate (0.5%) and mannitol (0.5 M, in AAP solution only) are added afterward. The enzyme solution is sterilized by passing through 0.45 μm filters. For *P. nereocystis* preprocessing, the thallus was cut into pieces of 4–5 cm^2^ and cleaned with Betadine (1%). For enzymatic treatment, the thallus pieces are transferred to PAP solution for 0.5–2 h with rotation (100 rpm). Then, the protoplasts are separated by 35 μm filtration followed by centrifugation at 500 rpm for 5 min. The protoplasts are washed and recentrifuged three times with f/2 medium and 0.2 M mannitol. The protoplast generation is confirmed by Calcofluor White ST (0.01%) or FDA [74].

*Symbiodinium* sp. (Symbiodiniaceae, Dinoflagellata) is a microalga in Dinophyta. Protoplasts of *Symbiodinium* are prepared enzymatically with cellulase. *Symbiodinium* sp. cells were grown in the medium composed of filtered seawater and Diago’s IMK at pH 7.9 with a 12 h:12 h lighting cycle at 50 µmol photons m^−2^ s^−1^. For cell wall digestion, cells in the log phase were collected by centrifugation at 2000× *g* for 5 min. The enzyme solution was prepared by dissolving 0.5 M D-sorbitol and 1.5 or 3 KU cellulase in 10 mL of culture medium. Cells were resuspended in the enzyme solution and incubated at 29–30 °C with rotation at 100 rpm for 36–48 h. Protoplasts were separated by centrifugation at 2000× *g* for 5 min and washed with 0.5 M D-sorbitol, 0.5 M sucrose, 25 mM CaCl_2_, and 100 μg/mL kanamycin dissolved in 10 mL of culture medium. Protoplasts were incubated at room temperature with shaking at 100 rpm for 3 h before the wash step was repeated again and were finally harvested by centrifugation at 200× *g* for 5 min. The protoplast generation was examined with Calcofluor White staining [75].

*Crypthecodinium cohnii* (Crypthecodiniaceae, Dinoflagellata) is a microalga in Dinophyta. Protoplasts were prepared with a chemical method using a cellulose synthesis inhibitor. Specifically, *C. cohnii* cells were grown in MLH liquid medium at 28°C in the dark. Exponentially growing cells were collected by centrifugation at 2500× *g* for 10 min and resuspended in 20% (*wt*/*vol*) Polyethylene glycol (PEG) by vortexing. The cells were spread on NLH agar plates supplemented with 0.4% (*wt*/*vol*) PEG 8000 and incubated at 28 °C in the dark for 48 h. For protoplast collection, the colonies were rinsed with fresh MLH medium and the eluant was retained. The step was repeated again and both the first and second eluants were purified through a 10-µm filter. The protoplast generation was confirmed by M2R [76].

Cyanobacteria are a group of prokaryotes capable of photosynthesis, which also have thick cell walls. Cyanobacterial species *Microcystis aeruginosa* (Microcystaceae, Cyanobacteria), *Anabaena flos-aquae* (Nostocaceae, Cyanobacteria), and *Anacystis nidulans* (Synechococcaceae, Cyanobacteria) were studied in protoplast preparation with an enzymatic method in the 1960s. Specifically, the cells of each species reaching the logarithmic phase of growth were collected and concentrated at 2000× *g* and washed twice with 0.5 M mannitol-0.03 M potassium phosphate buffer. The lytic solution was a 0.05% lysozyme solution. Protoplasts were generated by incubating the cells in the lytic solution for at least 4 h and collected by centrifugation. The resulting protoplasts were checked under phase contrast microscopes [53].

## 4. Conclusions

Life science once again is seeing another major advance in omics techniques, single-cell sequencing, after the first- and second-generation sequencing. However, the algal cell wall hinders the SCS technique application to algae. Fortunately, methods are available to make algal protoplasts from intact cells for the current SCS pipeline. Previous studies have established physical degradation, chemical chelating, and biological digestion of algal cell walls, among which biological digestion with chemical pretreatment has shown the greatest efficiency. Chemical chelators such as cation chelator EDTA can enhance enzymatic digestion by destabilizing the chemical bonds between cell wall components. Among the enzymes, cellulase and pectinase were proved to be the most effective as cellulose and pectin are the two major compositions of the algal cell wall. Macerozyme and driselase may enhance digestion. Proteinase, chitinase, and alginate lyase were used according to the chemical composition and structure of the algal cell wall. Therefore, various components of the cell wall of different types of algae require an effective combination of lytic enzymes in protoplast isolation. At the moment, existing methods still need to be optimized for different algal protoplast preparation for SCS analysis. As summarized in this review, green algae (Chlorophyta) among the four discussed phyla are the best studied in the cell wall structure and composition and have the most case studies of protoplast preparation. From these cases of all five phyla, it is clear that each phylum requires a different combination of lytic enzymes. Moreover, one critical drawback of such a method is that the room temperature required by the enzymes leads to mRNA degradation. The degradation of mRNA should be minimized by keeping cells at low temperatures, which eliminates the efficiency of the current enzymatic digestion protocol. Therefore, future efforts should focus on improving the physical or mechanical methods, using nanoparticles or glass beads accompanied with homogenization or sonication, and the enzymatic method using new enzymes extracted from benthic alga-predators such as plankton, crustaceans, and herbivorous fish.
